# External validation of the smartphone-based 6-minute walking test in patients with degenerative lumbar disorders undergoing epidural steroid injection

**DOI:** 10.1016/j.xnsj.2024.100561

**Published:** 2024-09-27

**Authors:** Michal Ziga, Martin N. Stienen, Anna Maria Zeitlberger, Stefanos Voglis, Luca Regli, Oliver Bozinov, Nicolai Maldaner

**Affiliations:** a Department of Neurosurgery, Kantonsspital St. Gallen & Medical School of St.Gallen, St. Gallen, Switzerland; b First Faculty of Medicine, Charles University in Prague, Prague, Czech Republic; c Spine Center of Eastern Switzerland, Kantonsspital St. Gallen & Medical School of St.Gallen, St. Gallen, Switzerland; d Department of Neurosurgery and Spine Surgery, Luzerner Kantonsspital and University of Lucerne, Lucerne, Switzerland; e Department of Neurosurgery, University Hospital Zurich & Clinical Neuroscience Centre, University of Zurich, Zurich, Switzerland

**Keywords:** 6-minute walking test, 6WT, External validity, Functional self-assessment, Objective functional impairment

## Abstract

**Background:**

The 6-minute walking test (6WT) has previously shown to be reliable and valid outcome measure in patients undergoing surgery for degenerative lumbar disorders (DLD). A role of 6WT in conservatively treated patients undergoing epidural steroid injection (ESI) remains unclear.

**Methods:**

About 50 patients with DLD, scheduled for ESI were assessed by the smartphone-based 6WT and common paper-based patient-reported outcome measures (PROMs), including the Core Outcome Measures Index [COMI] back, Oswestry Disability Index (ODI) and Short Form Survey (SF-12). Pearson correlation coefficient (PCC) was used to define the relationship between 6WT and PROMs. Reliability of the 6WT was determined by intraclass correlation coefficient (ICC). Age- and sex-adjusted objective functional impairment (OFI) z-scores were calculated for each patient.

**Results:**

A total of 50 patients (mean age 52 years, SD 13; 25% female), including 35 patients (70%) with lumbar disc herniation and 15 patients (30%) with lumbar spinal stenosis were included. The mean 6-minute walking distance (6WD) was 454.1 m (SD 89.1); the age- and sex-adjusted mean OFI z-score was −2.1 (SD 4.0). A total of 17 (34%) patients had mild, 8 (16%) moderate, and 4 (8%) severe OFI, while 21 (42%) had 6WT results within the normal population range (no OFI). The PCCs between the 6WD and VAS back pain were r=−0.30, ODI r=−0.43, COMI back r=−0.36, and PCS-12 r=0.51 (all p<.05). The ICC of the 6WT was β=0.92.

**Conclusions:**

This external validation in a patient cohort with DLD, which was managed conservatively, confirms the reliability and content validity of the 6WT with similar PCCs with PROMs compared to the original surgical cohort. The results confirm the 6WT as a valid and useful tool for assessing OFI in patients with DLD and a broad range of functional restrictions in an outpatient setting.

## Introduction

Lumbar degenerative disorders (DLD), such as lumbar disc herniation (LDH) or lumbar spinal stenosis (LSS), usually present with pain and/or neurological deficits that ultimately lead to functional impairment and a reduction in physical capacity [1,2]. Traditionally, patient reported outcome measures (PROMs) are deployed to determine the efficacy of an intervention in DLD [3,4]. But PROMs can be subject to inaccuracies and may not reflect a direct and objective measurement of functional impairment [2,5,6].

Several instruments, such as the self-paced walking test, the motorized treadmill test and the 6-minute walking test (6WT), have been used to assess walking capacity in patients with DLD. Among the available objective functional tests, the 6WT is one of the best studied and most widely used tools to assess objective functional impairment (OFI) in patients with DLD [[7], [8], [9], [10]].

Originally introduced in the field of internal medicine and already widely applied in the assessment of diseases like chronic obstructive pulmonary disease [11], the 6WT has more recently been studied and adopted as an objective measure of physical capacity in the field of spine surgery [10,[12], [13], [14], [15], [16]]. The desire for an easy-to-use objective tool to measure functional impairment before and after treatment for DLD led to various studies examining the 6WT. In their 2016 paper, Chan et al. first demonstrated a good correlation of the 6WT with the Tinetti score for standing and walking in a cohort of patients with LSS, and patients improved in performance at both 3 and 6 months after surgery [14]. Loske et al. analysed gait quality as a possible outcome measure after surgery for LSS, with 6WD as one of the secondary parameters showing improvement at both 10 weeks and 12 months postoperatively [15]. Takenaka et al. used a longitudinal analysis of the 6WD to determine the predictors (younger age, lower body weight, 1 level operative segment, decompression surgery, and better preoperative scores for trunk extensor strength and 6WD) that positively influence outcome 6 months after surgery [16]. Tosic et al. first demonstrated excellent test retest reliability (ICC=0.89) in a cohort of healthy subjects without known spine disease. Tosic et al. also provided 6WT normal population reference values that were later used to establish sex- and age-adjusted z-scores, which showed adequate content validity with commonly used PROMs [7,12,13,16].

We have recently developed and subsequently validated a smartphone app-based version of the 6WT, which allows patients to self-assess the maximum distance they can walk within 6 minutes (6WD=6-minute walking distance) [17,18]. In the cohort of patients undergoing surgical treatment for DLD, the 6WT was shown to be reliable and valid when compared to a range of commonly used patient-reported outcome measures (PROMs) [8,12,17]. The smartphone-based 6WT is convenient to administer in the patient's home environment and patients were found to prefer the test to completing paper-based PROMs [19].

However, the increasing acceptance of objective functional testing among DLD patients and surgeons raises the question of the generalizability of the 6WT to other DLD patient populations. For example, are the results of the 6WT also valid in patients treated conservative in an outpatient setting, as these patients represent a large proportion of the population suffering from symptomatic DLD? Little is known about the applicability of 6WT in patients scheduled for an epidural steroid injection (ESI) for DLD, although a small case series suggested that 6WT could be a useful tool for monitoring patient outcome and disability [18].

We hence set out to study the external content validity and reliability of the 6WT as a measure of OFI in cohort of patients prior to epidural steroid injection for DLD. Specifically, we examine the relationship of the 6WT to commonly used PROMs that were used in the 6WT's original validation study, as well as additional PROMs, that were not used in previous studies.

## Methods

### Patient inclusion

All adult patients with DLD, scheduled for elective epidural or transforaminal steroid injection in an outpatient setting between July 2020 and March 2022 for symptomatic (1) LDH or (2) LSS were prospectively screened for inclusion in the study at the Department of Neurosurgery of the Cantonal Hospital St. Gallen, Switzerland. Detailed inclusion and exclusion criteria are stated in the Supplemental Digital Content 1.

### The 6WT-app

The 6WT app (see Supplemental Fig. 1) is a smartphone app that uses Global Positioning System (GPS) coordinates to measure the maximum distance (in metres) that a subject can walk in 6 minutes (6WD) [8]. Both the distance walked and the time elapsed are continuously displayed on the screen as the 6WT is performed. With the aim of walking as fast as possible, the 6WD is the primary test outcome. Patients with DLD usually suffer from pain and functional impairment, which ultimately leads to less activity (e.g. slower walking, more breaks) and therefore less 6WD.

At the first consultation, patients were instructed on how to download and use the 6WT app. Instructions on how to perform the 6WT (on a level and straight path in their typical (home) environment) were given, as described previously.[18] The 6WT app is available free of charge for Apple iOS and Android at:

1. Google Play: https://play.google.com/store/apps/details?id=ch.webgearing.walkingapp&hl=de
2. Apple Store: https://apps.apple.com/de/app/6wt-app/id1454002232

### Data collection and patient reported outcome measures (PROMs)

Demographic and clinical data were collected at study inclusion. Both, subjective (PROMs) and objective (6WT) outcome assessments were performed prior to ESI following. The PROMs included Visual Analogue Scale (VAS) back and leg pain, the Oswestry Disability Index (ODI), the Core Outcome Measures Index (COMI) Back and the Short Form Survey (SF-12) with its 2 main score the Physical Component Summary (PCS-12) and Mental Component Summary (MCS-12) (see Supplemental Digital Content 1).

### Statistical considerations

Patient characteristics are expressed as mean ± standard deviation (SD) for continuous variables and count (percentage) for categorical variables. The results of the 6WT are presented as raw 6WD (in m) ± standard deviation (SD) and as standardized z-scores, adjusted for age and sex using reference values from the normal population [13]. In order to account for inter-individual differences in test scores, Z-scores express the number of SDs by which patients deviate from the mean of a spine-healthy reference population [9,13]. Moreover, as defined earlier [12], OFI z-scores were stratified into a 4-grade scale, according to the severity of functional impairment: Z-scores > -1 were classified as no OFI, ≤ −1 to −1.9 as mild OFI, ≤ −2 to −2.9 as moderate OFI, and ≤ −3 as severe OFI [13].

The relationship between 6WT scores and baseline PROMs was defined using Pearson correlation coefficient (PCC) r; values between 0 and 0.2 were interpreted as negligible, 0.3–0.4 as weak, 0.4–0.7 as moderate and 0.7–1 as strong relationship [20]. Intraclass correlation coefficient (ICC) was used to determine the agreement of repeated measures. The standard error of measurement (SEM) was calculated as the SD multiplied by the square root of 1 minus the intra-rater ICC [21,22]. Analyses were performed with IBM SPSS version 27.0.0.0 (IBM Corp., Armonk, NY, USA). p-values <.05 were considered significant.

## Results

### Patients’ demographics

Fifty consecutive patients undergoing epidural/transforaminal steroid injection for DLD were included in this study with a mean age of 52 years (SD 13). Detailed demographic and clinical variables of the study population are summarised in Table 1.

**Table 1:** Patients demographics.

	Study group
Age in years	52 (13)
Sex	
Male	25 (50%)
Female	25 (50%)
Body dimensions	
Height in cm	170.7 (16.0)
Weight in kg	77.4 (22.5)
BMI in kg/m^2^	25.2 (4.2)
Working status	
Full-time	35 (70%)
Part-time	3 (6%)
Retired	2 (4%)
Disabled	10 (20%)
Smoking status	
Smoker	5 (10%)
Nonsmoker	45 (90%)
ASA risk scale	
1	40 (80%)
2	6 (12%)
3	4 (8%)
Previous spine surgery	
Yes	12 (24%)
No	38 (76%)
	**N=50 (100%)**

Data is presented in count (%) or mean (standard deviation).

ASA, American Society of Anaesthesiologists risk scale; BMI, body mass index.

### Subjective PROMs and objective outcome measures

Table 2 shows pathology-specific characteristics. The most commonly affected level was L4/5 (50%) and 10% of patients presented with neurological deficit. Patients had mild to severe subjective pain and physical disability, as indicated by the VAS pain, ODI, COMI back and PCS-12. The study group had a relatively normal baseline mental health functioning (MCS-12: 45.0, SD 11.3).

**Table 2:** Clinical characteristics of the study group.

	Study group
Indication	
Lumbar Disc Herniation	35 (70%)
Lumbar Spinal Stenosis	15 (30%)
Lower Extremity Motor Deficit*	
M4	5 (10%)
M5	45 (90%)
Type of steroid injection	
Interlaminar epidural	7 (14%)
Transforaminal epidural	43 (86%)
Affected level	
L1/2	1 (2%)
L2/3	4 (8%)
L3/4	9 (18%)
L4/5	25 (50%)
L5/S1	11 (22%)
VAS pain measures	
Back	2.3 (2.9)
Leg	5.7 (2.0)
Oswestry Disability Index	30.4 (17.4)
Short Form 12	
Mental Component Score	45.0 (11.3)
Physical Component Score	37.3 (11.8)
Core Outcome Measure Index	
Back total score	5.4 (2.1)
Pain score	6.0 (1.9)
Disability score	4.1 (3.2)
	**N=50 (100%)**

Data is presented in count (%) or mean (standard deviation).

VAS, visual analogue scale.

⁎ According to the British Medical Research Council (BMRC) paresis grading.

**Fig. 1:**
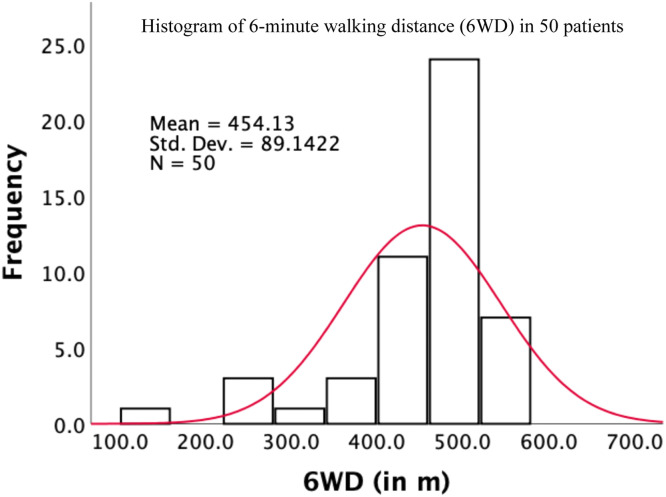
Histogram of 6-minute walking distance (6WD) in a sample of n=50 patients scheduled for epidural steroid injection for lumbar degenerative disc disease. The normal density curve is overlaid in red.

The mean 6WD was 454.1m (SD 89.1, Fig. 1), which translated into an OFI z-score of -2.1 (SD 4.0). According to the OFI severity stratification, 21 patients (42%) had no OFI, while 17 (34%), 8 (16%) and 4 (8%) patients had mild, moderate, and severe OFI, respectively (Table 3).

**Table 3:** 6-minute walking test (6WT) results presented as mean (standard deviation), OFI z-score and stratified into OFI categories.

6-minute walking test	Study group
6WD (m)	454.1 (89.1)
OFI, z-score	−2.1 (4.0)
OFI category	
No OFI	21 (42%)
Mild OFI	17 (34%)
Moderate OFI	8 (16%)
Severe OFI	4 (8%)
Total	**N=50 (100%)**

6WD, 6-minute walking distance; OFI, objective functional impairment.

### Test-retest reliability

The ICC for repeated 6WT measurements was excellent (β=0.92, 95% CI 0.87–0.96, p<.001), with a SEM of 25.2 m.

### Content validity

**Fig. 2:**
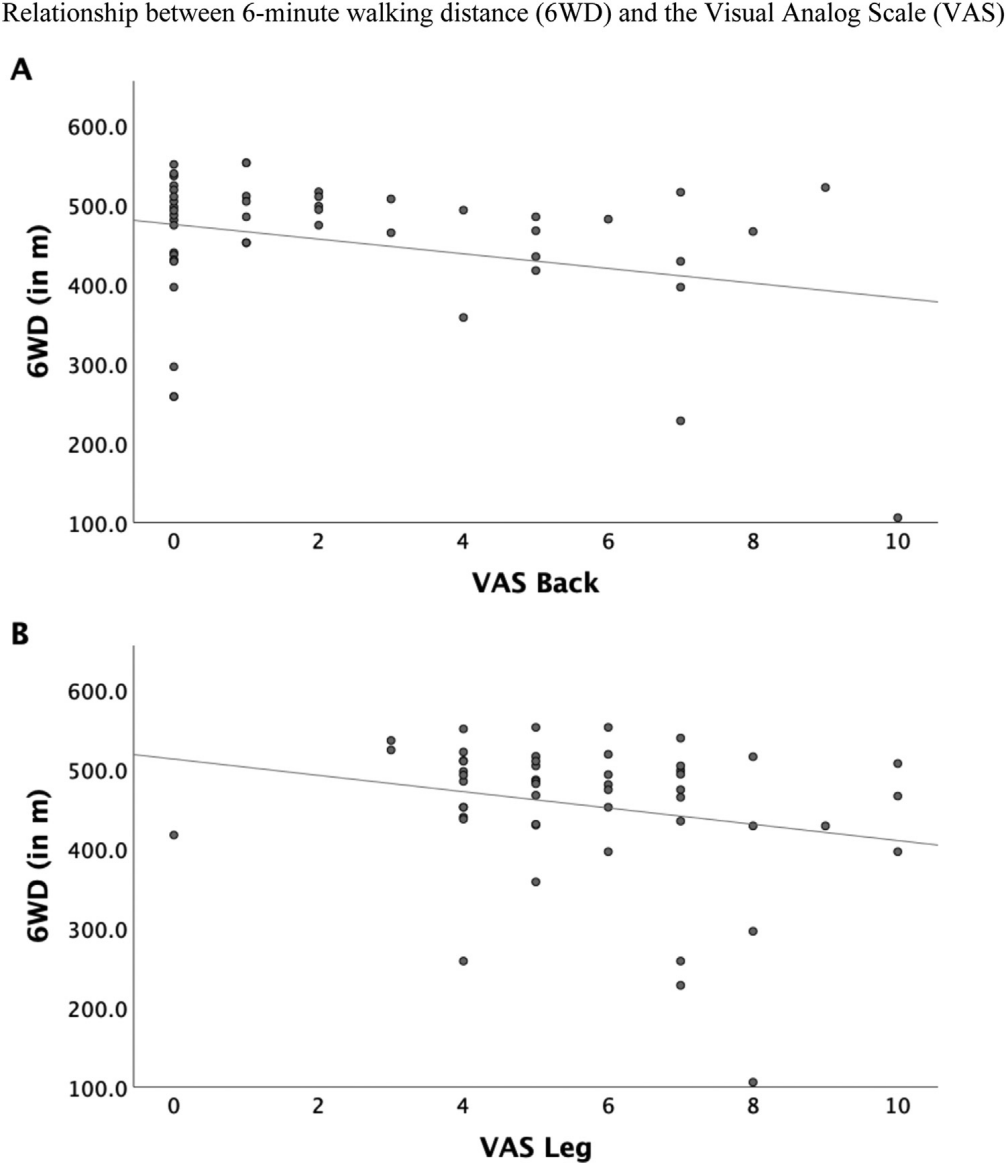
Relationship between 6-minute walking distance (6WD) and the Visual Analog Scale (VAS) for (A) back pain and (B) leg pain. Pearson's correlation coefficient (r) was -0.30 for VAS back pain and r=−0.23 for VAS leg pain.

**Fig. 3:**
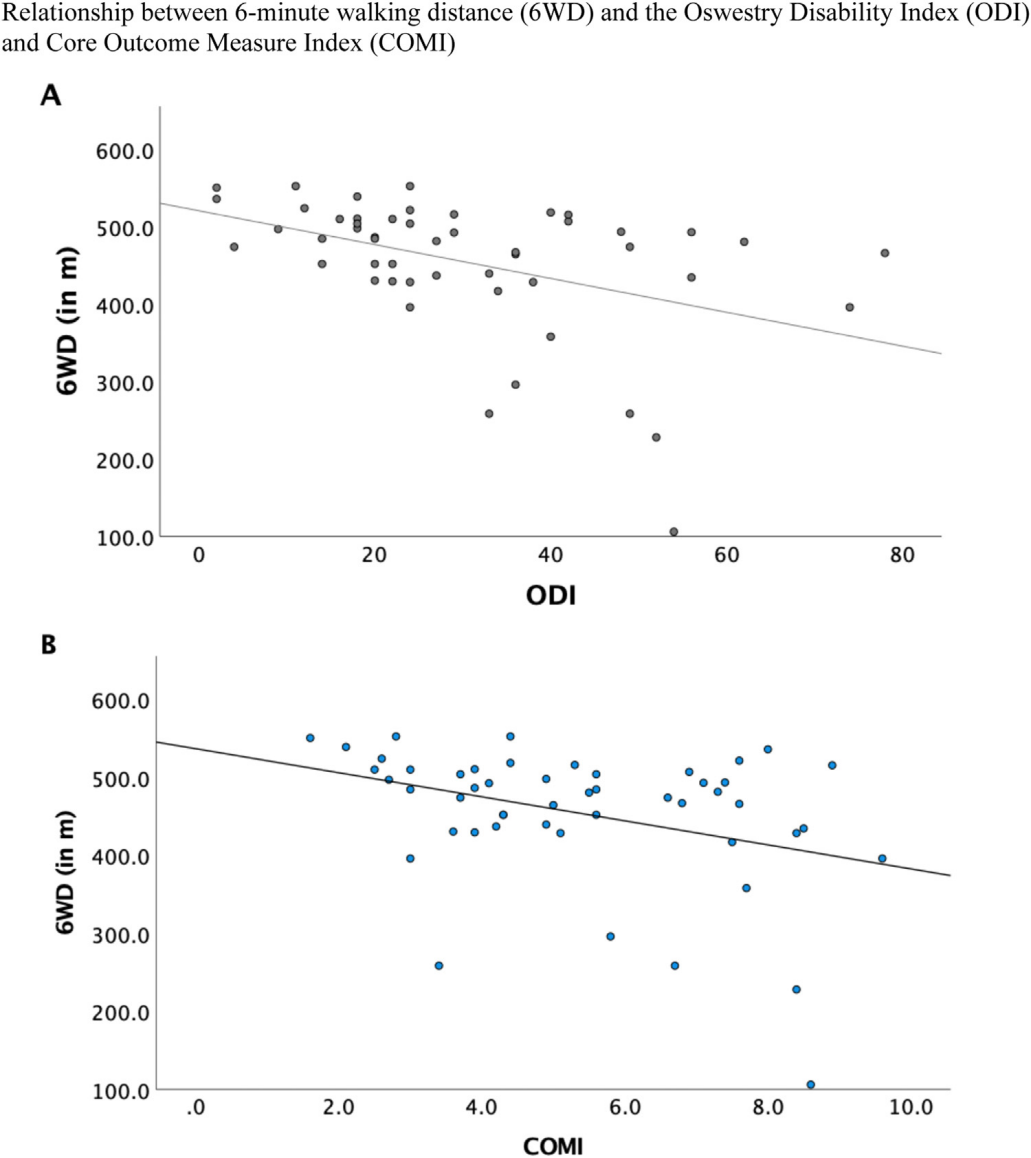
Relationship between 6-minute walking distance (6WD) and the Oswestry Disability Index (ODI), (A and B) Core Outcome Measure Index (COMI). Pearson's correlation coefficient (r) was −0.43 for ODI and r= −0.36 for COMI.

**Fig. 4:**
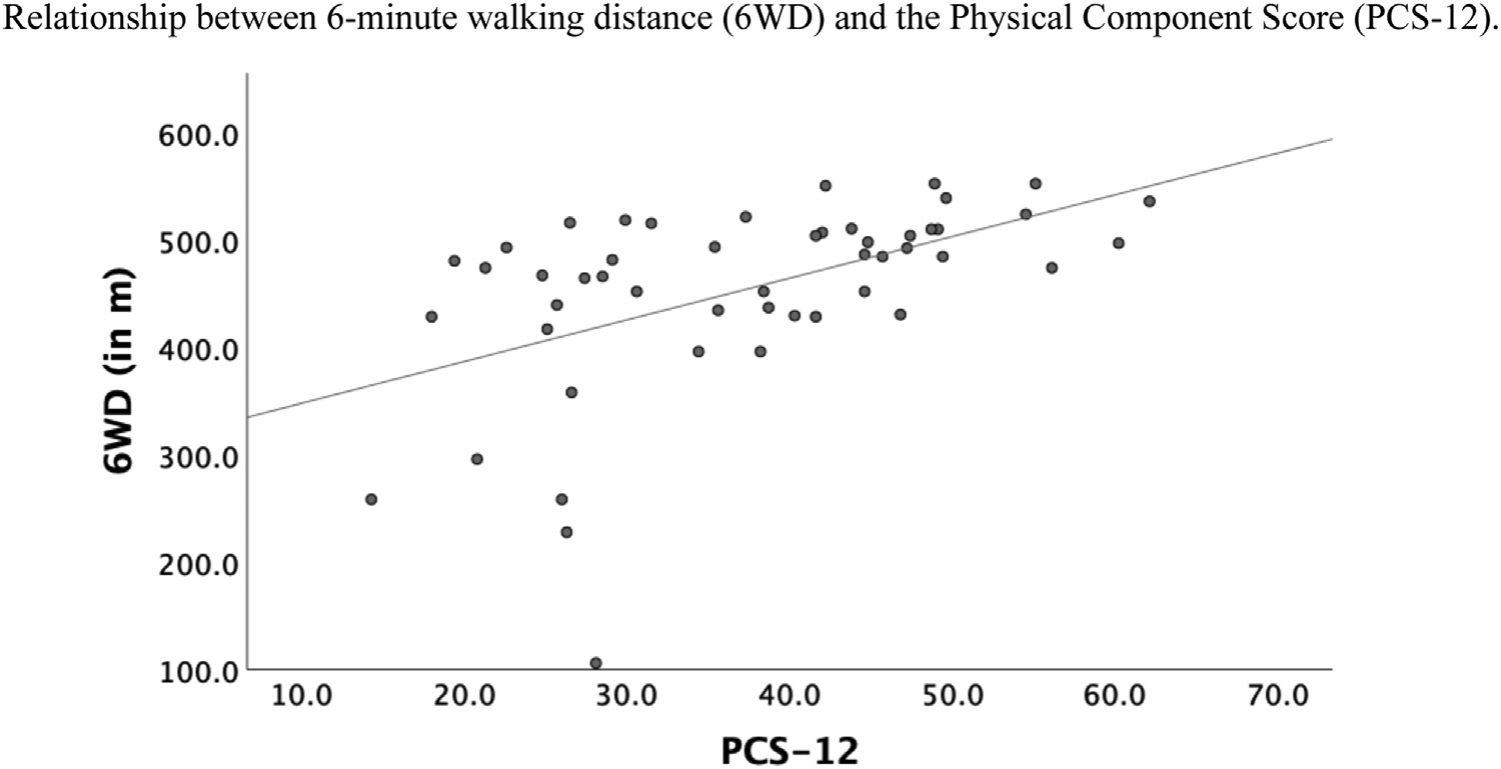
Relationship between 6-minute walking distance (6WD) and the Physical Component Score (PCS-12). Pearson's correlation coefficient for PCS-12 was r=0.51.

The 6WT showed significant negative correlations with the VAS back pain (r=−0.30) and VAS leg pain (r=−0.23), ODI (r=−0.43), COMI Back (r^=^−0.36) and a significant positive correlation with PCS-12 (r^=^0.51) (all p<.05; Table 4), indicating that patients with more disability and pain had less 6WD (Fig. 2, Fig. 3, Fig. 4). The correlation between 6WD and the MCS-12 mental health functioning subscale of the SF-12 was not significant.

**Table 4:** Pearson correlation of the 6WD with Patient Reported Outcome Measures prior to Epidural Steroid Injection.

	6WD	Z-Score	VAS Back	VAS Leg	ODI	COMI Back	PCS-12	MCS-12
6WD	1							
Z-Score	.34*	1						
VAS Back	−.30*	−.37*	1					
VAS Leg	−.23*	−.42*	.34*	1				
ODI	−.43*	−.48*	.51*	.58*	1			
COMI Back	−.36*	−.44*	.77*	.43*	−66*	1		
PCS-12	.51*	−.27	−.29v	−.30*	−.72*	−.49*	1	
MCS-12	.06	−.16	−.39*	−.17	−.43*	−.48*	.27	1

⁎ All p < 0.05 unless stated otherwise. 6WD, 6-minute walking distance; COMI, Core Outcome Measures Index; VAS, visual analogue scale; ODI, Oswestry Disability Index; PCS, Physical Component Score; MCS, Mental Component Score.

## Discussion

The aim of the study was to externally validate the 6WT in a cohort of conservatively managed patients prior to ESI in an outpatient setting. As prior studies determining OFI with the 6WT had exclusively included patients undergoing spine surgery [7,12,17,18], whereas conservatively treated patients represent the majority of all DLD patients, the question arose as to the feasibility and universality of the 6WT to detect OFI in this setting, and how test results correlate with a patient's own subjective perception of the disease. The patients in our cohort undergoing ESI represented a classic DLD sample [12]. Firstly, the 6WT showed excellent test-retest reliability (ICC 0.92). Secondly, the 6WD showed a significant correlation with the routinely used subjective PROMs. In addition to the PROMs that were used in the original study published in 2020 [12], the current validation study applied commonly used questionnaires (e.g., ODI and SF-12), which have not been used to cross-validate the 6WT and extend the current understanding of the relationship between the 6WT and PROMs [10,12,18].

### Reliability and content validity

The 6WT again showed excellent reliability, consistent with the previous published results in patients undergoing surgery, as well as in a cohort of subjects without known spinal disease [12,13]. This indicates that the test's main outcome 6WD provides consistent and reliable values when performed in short intervals.

Regarding content validity, PCCs for VAS back pain (previous cohort: r=−0.42; current cohort: r=−0.30) and VAS leg pain (previous cohort: r=−0.32; current cohort: r=−0.23) showed significant agreement, in line with the original surgical cohort [12]. Notably, PCC values were consistently slightly stronger in the surgical cohort compared to this nonoperative cohort. We can currently only speculate about possible reasons for this trend which might be due to lower overall 6WD values with higher standard deviation. Further research in larger patient cohorts need to investigate whether this is a statistical inaccuracy or not. The correlation coefficient indicated a particularly strong relationship between the 6WT and the PCS-12 (r=0.51, p<.001). This finding suggests that OFI as measured by the 6WT is relatively well represented by the Physical Component Score of the Short Form 12 and vice versa.

All linear relationships between the 6WT and PROM measures indicated that patients with more pain and disability had a shorter 6WD and a higher degree of OFI. It is important to note that the 6WT measures physical capacity, whereas PROMs are a subjective expression of how the patient feels. Patient's physical and mental health, perception of pain and disability and expectations about the outcomes of surgery may all influence PROM results. PROMs can therefore be subject to inaccuracies and may not reflect a direct and objective measure of functional impairment [2,5,6].

In contrast, no significant correlation was found between the SF-12 MCS and the 6WT. Interestingly, the study cohort was in good mental health, as represented by a relatively normal SF-12 MCS (45.0, SD 11.3). Prior works concentrating on objective functional testing in DLD patients scheduled for surgery noted slightly worse SF-12 MCS values (mean 43.9, SD 11.3) [9], and patients with worse functional status showed lower SF-12 MCS values (mean 44.3, SD 10.9 in n=230 patients without OFI versus mean 37.0, SD 10.9 in n=41 patients with severe OFI) [23] Considering this, our current data suggest that patients scheduled for ESI are generally in a better mental and physical condition than patients scheduled for surgery.

Overall, 6WT results showed robust correlations with all subjective measures of pain, functional disability, and health-related quality of life (hrQoL) (Table 4). This is in contrast to the Timed-Up and Go (TUG) or 5 Repetition-Sit-To-Stand (5R-STS) tests, which showed generally moderate correlations with most disability measures, but not as well with the VAS pain scales [9,24,25].

For the 6WT, a particularly strong correlation was seen with the VAS back pain and to a lesser extent with the VAS leg pain, suggesting that LBP and radiating leg pain adequately influence the 6WD. Nevertheless, similar to previous findings, none of the correlations between 6WD and PROMs in our study exceeded r=0.50, which supports the conclusion that the 6WT is an additional tool in the outcome assessment of spinal patients and should not replace but complement the well-established PROM questionnaires [12]. This notion gain increasing popularity in the academic community, and novel outcome scores that contain both subjective (PROMs) and objective information have recently been proposed [26].

### Stratification of objective functional impairment

Stratification of OFI is reflected by the z-score, which expresses the severity of disability, taking into account factors (age and gender) that may naturally influence physical capacity [9]. According to the baseline stratification of OFI severity, 21 patients (42%) had no OFI, while 17 (34%), 8 (16%) and 4 (8%) patients had mild, moderate and severe OFI, respectively.

When comparing the surgical (prior publication) and the conservative (current) cohort, only about a quarter of patients in the group scheduled for ESI had moderate to severe OFI, whereas almost two-thirds of patients in the surgical cohort presented with the same OFI severity [12]. The fact that DLD patients planned for an ESI instead of surgery present with lower levels of OFI is arguably to be expected. Together with the lower levels of subjective disability as expressed by PROMs, this finding, however, supports the credibility and generalizability of the results.

Our current study adds to previous findings in that it externally validates the 6WT psychometric properties in a cohort of conservatively managed patients prior to ESI. In contrast to other groups, we specifically built and applied a smartphone-based version of the 6WT that allows patients to self-assess their physical capacity in an outpatient setting. In times where healthcare spending is increasing worldwide and hospital resources are limited, to be able to let patient assess their functional impairment in their home environment represents an important advantage which may increase test adoption from both physicians as well as patients. Our study confirms that the smartphone-based 6WT is reliable, valid and easy-to-use when assessing spine patients in an outpatient setting.

### Strengths and limitations

The strengths of the study are the prospective design and the predefined protocol, which includes both the common subjective PROMs and the validated 6WT physical performance test [12]. Our sample size of 50 patients can be considered small and may have influenced the results. However, it is unlikely that a larger sample size would have changed the results, as we show consistent and significant correlations of the 6WT with PROMs, similar to the original study.

Furthermore, our results need to be interpreted in the context of our study cohort of DLD patients with both LDH and LSS, which is consistent with previous studies [12]. In addition, we excluded patient with other medical reasons interfering with the patient's ability to walk and perform the 6WT. While we set this exclusion criteria to minimize bias on the 6WT results it might have limited the generalizability of the study as many of patients presenting with lower back pain may have other joint complaints or systemic diseases that impact their 6WT and may introduce a confounder. Lastly, 24% of patient in our cohort had prior spine surgeries which were either related or unrelated to the current problem that was then treated with injection. However, in all cases that were related and/or addressed the same level prior surgery was at least 6 months before the injection. This was to ensure that patients had adequate time to recover from prior surgery so that change in subjective and objective outcome could reliably be attributed to the injection. However, future works focusing on either subgroups with a larger sample size may shed light on potential functional differences between the 2 pathologies.

## Conclusion

This external validation in a conservatively managed cohort of patients with DLD scheduled for ESI confirms the reliability and content validity of the 6WT with similar Pearson correlations with PROMs compared to the original report. The results support the 6WT as a valid and useful tool for assessing OFI in patients with DLD in an outpatient setting.

## Ethical considerations

The study was approved by the local ethic committee (Kantonale Ethikkommission, EKOS – 2019-01209) and was registered as NCT04062942 at http://clinicaltrials.gov. All patients provided written informed consent prior to study inclusion.

## Ethical approval

All procedures performed in studies involving human participants were in accordance with the ethical standards of the institutional and/or national research committee and with the 1964 Helsinki declaration and its later amendments or comparable ethical standards.

## Declaration of generative AI and AI-assisted technologies in the writing process

During the preparation of this work the authors used ChatGPT in order to improve the readability and language of the manuscript. After using this tool/service, the authors reviewed and edited the content as needed and take full responsibility for the content of the published article.

## Declaration of competing interest

All authors certify that they have no affiliations with or involvement in any organization or entity with any financial interest (such as honoraria; educational grants; participation in speakers' bureaus; membership, employment, consultancies, stock ownership, or other equity interest; and expert testimony or patent-licensing arrangements), or nonfinancial interest (such as personal or professional relationships, affiliations, knowledge or beliefs) in the subject matter or materials discussed in this manuscript.

## Appendix Supplementary materials

Image, application 1

Image, application 2
