# Supplementary material for: External validation of the smartphone-based 6-minute walking test in patients with degenerative lumbar disorders undergoing epidural steroid injection
Source: N Am Spine Soc J. 2024 Sep 27;20:100561. doi: 10.1016/j.xnsj.2024.100561 (PMC11546260; doi:10.1016/j.xnsj.2024.100561)
Supplement: Supplementary file 1 [file mmc1.docx]

**Supplemental Digital Content 1.**

**1. Inclusion and Exclusion Criteria for the study cohort**

Patients fulfilling all of the inclusion criteria were considered for this study:

• Patients with DLD not primarily treated surgically but scheduled for elective epidural or transforaminal steroid injection in an outpatient setting with (1) lumbar disc herniation (LDH) or (2) lumbar spinal stenosis (LSS)

• Male and Female subjects ≥ 18 years

• Written informed consent.

Patients were not enrolled if any of the following exclusion criteria were met:

- Pregnancy
- Inability to walk (extreme pain or severe neurological deficits)
- Severe heart failure corresponding to > NYHA III
- Lung diseases (e.g. lung cancer, diffuse parenchymal lung disease, severe chronic obstructive lung disease (COPD) corresponding to > Gold III)
- Other medical reasons interfering with the patient’s ability to walk and perform the 6WT (e.g. osteoarthritis disease of the lower extremities, Parkinson’s disease, hip or knee prosthesis, peripheral artery disease causing intermittent claudication, etc.)
- Inability to complete assessment (planning to move, no smartphone, etc.)

**2. Detailed description of Patient Reported Outcome Measures (PROMs) used in this study**

1. The Visual Analogue Scale (VAS) measured for lower back pain (back) and lower extremity (leg) pain (ranging from 0 (none) to 10 (severe pain))
2. The Oswestry Disability Index (ODI) (range: 0% (minimal disability) to 100% (bedbound or exaggerating))
3. The Core Outcome Measures Index (COMI) Back with one main and two subscales
4. COMI Back (range: 0 (none) to 10 (severe back-related disability))
5. COMI Subscale pain intensity (range: 0 (none) to 10 (severe pain))
6. COMI Subscale disability (range: 0 (none) to 10 (severe disability))
7. Short Form Survey (SF-12) with its two main scores:
8. Physical Component Summary (PCS-12, scores >50 indicate a better-than-average health-related quality of life, while scores <50 suggest below-average health)
9. Mental Component Summary (MCS-12, scores >50 indicate a better-than-average health-related quality of life, while scores <50 suggest below-average health)
