# Supplementary material for: External validation of the smartphone-based 6-minute walking test in patients with degenerative lumbar disorders undergoing epidural steroid injection
Source: N Am Spine Soc J. 2024 Sep 27;20:100561. doi: 10.1016/j.xnsj.2024.100561 (PMC11546260; doi:10.1016/j.xnsj.2024.100561)

**Supplemental Figure 1**

Screenshot of the 6-minute Walking Test smartphone application (“6WT” app) depicting the beginning of the test which currently is at 0:21 minutes and 16 m. The main outcome of the app is the six-minute walking distance (6WD) which can be converted into a standardized age- and sex-adjusted z-scores. The patients z-score in this case would be -7.1, which represents severe objective functional impairment (OFI) according to our previous research (*Tosic et al.: Normative data of a smartphone app-based 6-minute walking test, test-retest reliability, and content validity with patient-reported outcome measures. J Neurosurg Spine. 2020 May 29:1-10.doi:10.3171/2020.3.SPINE2084. Epub ahead of print. PMID: 32470938.*).


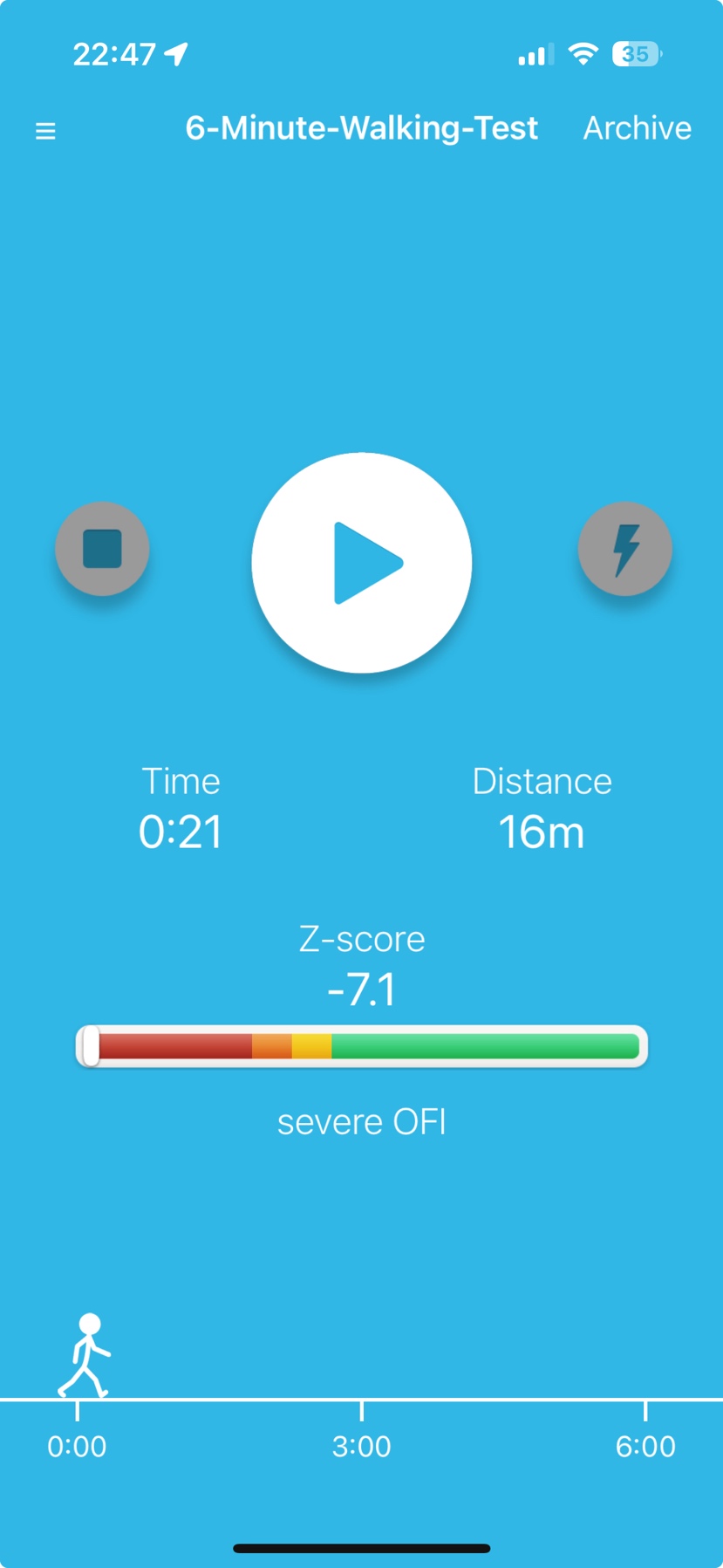

Supplement: Supplementary file 2 [file mmc2.docx]
